# Insulin resistance–related metabolic burden, white matter microstructure, and cognitive change: the HABS-HD study

**DOI:** 10.3389/fnins.2026.1967890

**Published:** 2026-10-01

**Authors:** Huaiyi Li, Zhiliang Tian, Xiaoqian Wang, Weiwei Gao, Jingjing She, The Health and Aging Brain Study (HABS-HD) Study Team, Sid E O’Bryant, Kristine Yaffe, Arthur Toga, Robert Rissman, Leigh Johnson, Meredith Braskie, Kevin King, James R Hall, Melissa Petersen, Raymond Palmer, Robert Barber, Yonggang Shi, Fan Zhang, Rajeh Nandy, Roderick McColl, David Mason, Bradley Christian, Nicole Phillips, Stephanie Large, Joe Lee, Badri Vardarajan, Monica Rivera Mindt, Amrita Cheema, Lisa Barnes, Mark Mapstone, Annie Cohen, Amy Kind, Ozioma Okonkwo, Raul Vintimilla, Zhengyang Zhou, Michael Donohue, Rema Raman, Matthew Borzage, Michelle Mielke, Beau Ances, Ganesh Babulal, Jorge Llibre-Guerra, Carl Hill, Rocky Vig

**Affiliations:** 1 Department of Radiology, The Second Affiliated Hospital of Xiamen Medical College, Xiamen, China; 2 Department of Hepatobiliary and Pancreatic Surgery, The First Affiliated Hospital of Hunan University of Medicine, Huaihua, China; 3 Department of Cardiology, Xiamen Humanity Hospital, Fujian Medical University, Xiamen, Fujian, China; 4 Department of Neurology, Zhongshan Hospital of Xiamen University, School of Medicine, Xiamen University, Xiamen, China; 5 Department of Radiology, The First Affiliated Hospital of Hunan University of Medicine, Huaihua, China

**Keywords:** cognitive decline, diffusion tensor imaging, insulin resistance, radial diffusivity, triglyceride-glucose index, white matter microstructure

## Abstract

**Background:**

Insulin resistance–related metabolic burden is associated with cognitive aging, but the relevant white matter microstructural features remain unclear. We examined statistical indirect associations between metabolic indices and cognition through tract-level white matter measures, including associations with subsequent cognitive change.

**Methods:**

Cross-sectional and longitudinal analyses used Health and Aging Brain Study–Health Disparities (HABS-HD) data. Exposures were the triglyceride–glucose (TyG) index and its composites with waist-to-height ratio (TyG-WHtR) and visceral adiposity index (TyG-VAI). Candidate mediators were axial and radial diffusivity (RD) in 32 white matter tracts; outcomes covered six cognitive domains. High-dimensional mediation analysis with within-model false discovery rate (FDR) correction selected candidate pathways, supplemented by model-level multiplicity analyses. Selection stability was assessed using 1,000 participant-level bootstrap samples. All candidates underwent longitudinal mixed-effects analysis, with indirect estimates calculated as baseline exposure–white matter coefficients multiplied by white matter-by-time coefficients. Intervals used 2,000 participant-level cluster bootstrap samples. Sensitivity analyses adjusted for white matter hyperintensity (WMH) volume and covariate-by-time interactions.

**Results:**

Of 2,606 participants, 788 had two cognitive assessments a median of 2.17 years apart. Fourteen candidate pathways were selected, all involving RD and TyG or TyG-WHtR; 11 had 95% conditional Monte Carlo uncertainty intervals excluding zero. Two of 36 models met the supplementary model-level FDR criterion. Five longitudinal pathways had negative indirect estimates with 95% bootstrap intervals excluding zero: four involved right cingulum RD and processing speed or executive function, and one involved right superior corona radiata RD and processing speed. Four also met the FDR criterion for RD-by-time interactions. The TyG-WHtR–right cingulum RD–executive function indirect estimate was -0.0054 per year (95% bootstrap interval, -0.0110 to -0.0009). Selection frequencies ranged from 6.7% to 42.0%. After WMH and WMH-by-time adjustment, intervals excluded zero for two of these five pathways, decreasing to one with additional core covariate-by-time interactions.

**Conclusion:**

Triglyceride–glucose index and TyG-WHtR showed statistical indirect associations with cognition through white matter RD, with some pathways associated with subsequent cognitive change. Limited selection stability and sensitivity to WMH and covariate-by-time adjustment warrant evaluation in independent cohorts.

## 1 Introduction

Insulin resistance (IR) is a common, modifiable metabolic abnormality in middle-aged and older adults and a key contributor to the pathophysiology of type 2 diabetes and cardiovascular disease (Tahapary et al., 2022). The hyperinsulinemic–euglycemic clamp is the gold standard for assessing insulin sensitivity, but its use in large cohorts is limited by practical constraints. The triglyceride–glucose (TyG) index, calculated from fasting triglyceride and glucose concentrations, is therefore widely used as a surrogate measure of IR (Tahapary et al., 2022). Combining TyG with the waist-to-height ratio (WHtR) or visceral adiposity index (VAI) yields the composite indices TyG-WHtR and TyG-VAI, respectively. These indices incorporate additional information on central adiposity and metabolic features related to visceral fat (Amato et al., 2010). Population-based studies have linked these indices to cognitive health. A meta-analysis of 12 observational studies reported an association between higher TyG and adverse cognitive outcomes (Liu et al., 2024). A cohort study of 8,946 middle-aged and older adults found a dose–response relationship between TyG-WHtR and the risk of incident cognitive impairment (Hou et al., 2024). However, the brain structural features involved in these associations remain incompletely understood.

White matter microstructure may contribute to the association between metabolic burden and cognitive aging. Oligodendrocytes require a continuous supply of energy and lipids to synthesize and maintain myelin. IR-related disturbances in glucose and lipid metabolism, together with vascular dysfunction, may disrupt white matter homeostasis (García-García et al., 2022; Tepavčević, 2021). Diffusion tensor imaging (DTI) enables *in vivo* assessment of white matter microstructure by quantifying the directional diffusion of water molecules. Studies of healthy aging have associated poorer white matter microstructural integrity with slower processing speed. Longitudinal changes in white matter diffusion measures have also been coupled with changes in processing speed (Oschwald et al., 2019; Penke et al., 2010). Population-based studies have also linked IR and TyG-related indices to diffusion abnormalities in the corpus callosum, corona radiata, and internal capsule, and to greater white matter hyperintensity (WMH) burden (Ryu et al., 2014; Shu et al., 2025; Zhou et al., 2024). These findings support examining metabolic burden, white matter microstructure, and cognition together.

Most existing evidence concerns pairwise associations among these variables. Few studies have directly examined statistical indirect associations between metabolic burden and cognition involving white matter imaging measures. Small cross-sectional clinical studies in people with type 2 diabetes or cerebral small vessel disease have examined corpus callosum fractional anisotropy (FA) and perivascular diffusion measures (Li et al., 2026; Wang X. et al., 2022; Yang et al., 2025). These phenotypes capture different diffusion characteristics but do not adequately characterize how associations are distributed across white matter tracts and cognitive domains. Evaluating multiple IR-related metabolic indices, tract-level DTI measures, and cognitive domains in a large community cohort would extend this evidence. Longitudinal follow-up could then assess whether these baseline candidate pathways are associated with subsequent cognitive change.

Using the large, multiethnic community sample of the Health and Aging Brain Study–Health Disparities (HABS-HD; Petersen et al., 2025), we examined associations of TyG, TyG-WHtR, and TyG-VAI with six cognitive domains. We assessed indirect associations involving axial diffusivity (AD) and radial diffusivity (RD) across 32 white matter tracts. High-dimensional mediation analysis (HIMA) was used to identify candidate pathways in the cross-sectional analysis. These pathways were then evaluated in relation to subsequent cognitive change among participants with two cognitive assessments. Exploratory moderation analyses examined whether these indirect associations differed by sex, apolipoprotein E (APOE) ε4 carrier status, or race and ethnicity. We hypothesized that indirect associations involving white matter microstructure would link higher IR-related metabolic burden to poorer cognitive performance. We further hypothesized that some baseline candidate pathways would be associated with faster subsequent cognitive decline.

## 2 Materials and methods

### 2.1 Study design and participants

We used HABS-HD data to conduct a baseline cross-sectional analysis and a longitudinal analysis of subsequent cognitive change. HABS-HD is an ongoing community-based cohort study designed to characterize brain aging, cognitive health, and health disparities among Mexican American, non-Hispanic Black, and non-Hispanic White populations. HABS-HD was approved by the University of North Texas Health Science Center Institutional Review Board and was conducted in accordance with the Declaration of Helsinki. All participants or their legally authorized representatives provided written informed consent. This study used deidentified data from HABS-HD Releases 7 and 8 (Petersen et al., 2025).

Baseline was defined as each participant’s earliest available HABS-HD clinical visit. Eligibility required complete data for calculating all three IR-related metabolic indices, constructing all baseline cognitive domain scores, and adjusting for the core covariates. A DTI scan obtained within 90 days before or after the baseline clinical visit was also required for inclusion. DTI quality-control criteria required FA values between 0 and 1, positive AD, RD, and mean diffusivity (MD) values, and complete tract-level DTI measures. The final cross-sectional sample included 2,606 participants, of whom 788 had two eligible cognitive assessments and formed the longitudinal sample. Participant selection is shown in Figure 1.

**FIGURE 1:**
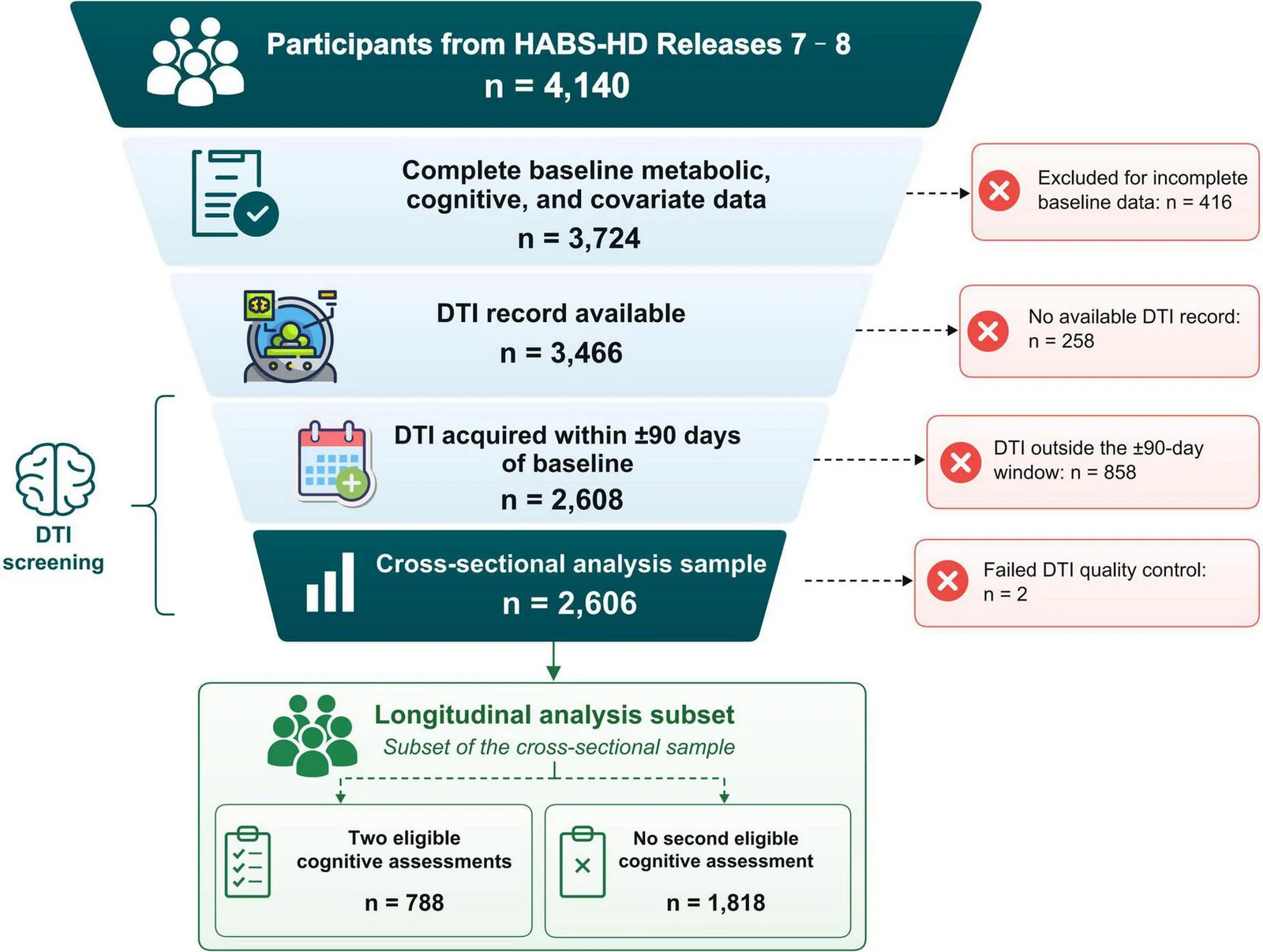
Participant selection for the cross-sectional and longitudinal analyses. Participants were selected from HABS-HD data releases 7–8. The cross-sectional sample comprised 2,606 participants with complete baseline data and quality-controlled DTI acquired within ± 90 days of the baseline clinical visit. Of these, 788 with two eligible cognitive assessments formed the longitudinal subset. DTI, diffusion tensor imaging; HABS-HD, Health and Aging Brain Study–Health Disparities.

### 2.2 Insulin-resistance surrogate indices

Fasting venous blood samples were collected at the baseline clinical visit (Petersen et al., 2025). Fasting glucose, triglyceride, and high-density lipoprotein cholesterol (HDL-C) concentrations were obtained from clinical laboratory measurements. Waist circumference, height, and body mass index (BMI) were obtained from standardized physical measurements. Three surrogate indices were used to characterize IR-related metabolic burden (Tahapary et al., 2022):


TyG=ln⁢[triglycerides⁢(mg/dL)×glucose⁢(mg/dL)/2];

WHtR=waist⁢circumference/height;

TyG⁢-⁢WHtR=TyG×WHtR;

TyG⁢-⁢VAI=TyG×VAI.

VAI was calculated using published sex-specific formulas (Amato et al., 2010):


Men:VAI=[waistcircumference(cm)/(39.68+1.88×BMI)]×

[triglycerides⁢(mmol/L)/1.03]×[1.31/HDL⁢-⁢C⁢(mmol/L)];

Women:VAI=[waistcircumference(cm)/(36.58+1.89×

BMI)]×[triglycerides(mmol/L)/0.81]×

[1.52/HDL⁢-⁢C⁢(mmol/L)].

### 2.3 Diffusion MRI acquisition and processing

Magnetic resonance imaging (MRI) was performed on 3 T Siemens scanners, including Skyra E11c and Magnetom Vida (Petersen et al., 2025). HABS-HD updated the Vida acquisition protocol to Vida2 on August 1, 2024. DTI scans included in this study were acquired between March 22, 2017, and July 31, 2024; therefore, only the Skyra and Vida1 protocols were represented.

DTI was acquired using an axial single-shot echo-planar imaging sequence with simultaneous multislice acceleration. Acquisition parameters included a repetition time/echo time of 4,000/92 ms, a voxel size of 1.7 mm × 1.7 mm × 2.5 mm, and a flip angle of 90°. The sequence included 64 diffusion-encoding directions with a b value of 1,000 s/mm^2^.

Diffusion MRI processing and quality control were performed by the HABS-HD imaging team at the University of Southern California. Data underwent distortion correction using the FMRIB Software Library (Smith et al., 2004), after which FA, AD, MD, and RD were calculated. Mean values were extracted from 32 tract-level white matter regions of interest using a DTI atlas (Oishi et al., 2009), yielding 128 measures. According to the HABS-HD image release criteria, scans were excluded if diffusion gradient information was missing, information was inconsistent with other imaging modalities, or any processing step failed, including distortion correction, tensor-based feature extraction, or atlas registration for region-of-interest definition. The released diffusion summaries contained measurements from analyses that passed the HABS-HD quality-control standards.

For this study, we used these released tract-level measures and additionally checked their completeness and numerical ranges before harmonization. FA values were required to lie between 0 and 1, AD, MD, and RD values were required to be positive, and all 128 measures were required to be nonmissing. These checks excluded two of the 2,608 participants with DTI acquired within 90 days of the baseline clinical visit, leaving 2,606 participants. ComBat harmonization was then applied to reduce between-scanner differences, with scanner specified as the batch variable and age and sex effects preserved (Fortin et al., 2017). Harmonized DTI measures were converted to z scores.

Axial diffusivity and RD were selected as the primary DTI mediator measures to examine diffusion along the principal diffusion axis and mean diffusion perpendicular to that axis, respectively. MRI–histology studies indicate that some tensor-derived measures are associated with myelin-related tissue characteristics, although these measures are not specific to a particular tissue pathology (Lazari and Lipp, 2021). FA is a normalized summary measure derived from the three tensor eigenvalues and can reflect different combinations of axial and radial diffusion changes. MD is the mean of the three eigenvalues and, on the original tensor-derived scale, satisfies MD = (AD + 2RD)/3. FA and MD were therefore excluded from the primary high-dimensional mediator screening to reduce redundant testing of related tensor-derived measures and limit the multiple-testing burden.

### 2.4 Neuropsychological assessment and cognitive domain scores

Cognition was assessed using standardized neuropsychological tests. These included the Trail Making Test Parts A and B, Digit Symbol Substitution Test, Digit Span Forward and Backward, and Spanish–English Verbal Learning Test (SEVLT) delayed recall. The battery also included Wechsler Memory Scale Logical Memory Story A immediate and delayed recall, letter fluency (F, A, and S), Animal Naming, and the Mini-Mental State Examination. Because the Trail Making Test Parts A and B were scored by completion time, their scores were reversed before standardization so that higher z scores consistently indicated better performance.

The cross-sectional analysis used baseline cognitive data. Each test score was converted to a z score using its baseline mean and standard deviation in the final cross-sectional sample. Six cognitive domain scores were then constructed. Processing speed was the mean of the reversed Trail Making Test Part A and Digit Symbol Substitution Test z scores. Executive function was represented by the reversed Trail Making Test Part B z score. Working memory was the mean of the Digit Span Forward and Backward z scores. Episodic memory was the mean of the SEVLT delayed recall and Logical Memory Story A immediate and delayed recall z scores. Verbal fluency was the mean of the FAS letter fluency and Animal Naming z scores. Global cognition was represented by the Mini-Mental State Examination z score.

The longitudinal analysis used cognitive data from the first and second visits. Each test was standardized using the mean and standard deviation estimated from the full cross-sectional baseline sample, with the same parameters applied to both visits. Longitudinal domain scores used the same test combinations and scoring directions as the cross-sectional scores. Time was defined as the number of years between each cognitive assessment and the participant’s baseline cognitive visit.

### 2.5 Covariates and additional measures

Core covariates included age, sex, years of education, race and ethnicity, hypertension, current smoking status, and MRI scanner. Race and ethnicity were modeled as Hispanic, non-Hispanic Black, and non-Hispanic White, with non-Hispanic White as the reference category. Hypertension was defined by a history of hypertension or elevated blood pressure on repeated measurements. Diabetes was defined by glycated hemoglobin (HbA1c) ≥ 6.5% or a history of diabetes. Participants with a documented previous stroke in the clinical records were classified as having a history of stroke.

Apolipoprotein E ε4 carrier status was determined from rs429358 and rs7412 genotypes. DNA was extracted from buffy coat samples, and APOE genotyping was performed using TaqMan real-time polymerase chain reaction (PCR; Petersen et al., 2025). Participants with at least one ε4 allele were classified as APOE ε4 carriers; all others were classified as noncarriers.

WMH volume was quantified from fluid-attenuated inversion recovery (FLAIR) images using the lesion growth algorithm in the Lesion Segmentation Toolbox for Statistical Parametric Mapping (Schmidt et al., 2012). Only data that passed quality control for both the FLAIR images and WMH lesion maps were used. Among eligible WMH measurements obtained within 90 days before or after the baseline clinical visit, the measurement closest to baseline was selected. Raw WMH volume was expressed in mL and transformed as ln(WMH volume + 1) for analysis.

### 2.6 Statistical analysis

All analyses were performed using R version 4.6.0. Statistical tests were two-sided, with a significance level of 0.05. Continuous variables were summarized as mean [standard deviation (SD)] or median [interquartile range (IQR)], depending on their distribution; categorical variables were summarized as counts (percentages). Pearson correlations were calculated between the three IR surrogate indices and six cognitive domains. Across all 18 tests, the Benjamini–Hochberg (BH) procedure for false discovery rate (FDR) control and the Bonferroni correction were applied separately.

The primary cross-sectional analysis used HIMA to assess statistical indirect associations involving tract-level DTI measures. Exposures were z-standardized TyG, TyG-WHtR, and TyG-VAI, and outcomes were the six baseline cognitive domain scores. AD and RD were modeled separately, with 32 white matter tracts evaluated for each measure, yielding 36 models. Models were adjusted for age, sex, education, race and ethnicity, hypertension, current smoking status, and scanner. Within each model, the 32 *P*-values from the distribution-of-the-composite-null (DACT) test were adjusted using BH-FDR. Pathways with *q* ≤ 0.05 were designated as HIMA-selected candidate pathways. The indirect effect was defined as the product of the exposure–DTI coefficient (a path) and the DTI–cognition coefficient (b path), denoted a × b. For candidate pathways, 20,000 Monte Carlo simulations were used to estimate 95% uncertainty intervals conditional on the selection results in the original sample. These intervals did not account for additional uncertainty arising from mediator selection.

Cross-model multiplicity was assessed in a supplementary analysis by applying BH-FDR to the Simes combined *P*-values from the 36 models. Each Simes *P*-value was calculated from the corresponding model’s 32 DACT *P*-values before BH adjustment. This analysis was not used as an additional criterion for pathway selection. HIMA selection stability was evaluated using 1,000 participant-level nonparametric bootstrap samples, with all 36 models rerun in each sample. Selection frequencies were reported for the original candidate pathways, without applying a *post hoc* stability threshold.

All cross-sectionally selected candidate pathways were carried forward into the longitudinal follow-up analysis without mediator reselection. The a path was re-estimated among participants with two eligible cognitive assessments who were included in the corresponding longitudinal model. Cognitive change was analyzed using linear mixed-effects models fitted by maximum likelihood. Models included time, baseline exposure, baseline DTI mediator, time-by-exposure and time-by-DTI mediator interactions, core covariates, and a participant-specific random intercept. The longitudinal indirect estimate was defined as a × b_time, where b_time was the coefficient for the time-by-DTI mediator interaction. A participant-level cluster bootstrap with 2,000 resamples jointly re-estimated a and b_time and yielded 95% percentile intervals. An interval excluding zero was the primary descriptive criterion for longitudinal support. *P*-values for b_time were adjusted using BH-FDR across all carried-forward pathways, providing supplementary evidence for the component path.

Exploratory moderation analyses examined whether candidate pathways differed by sex, APOE ε4 carrier status, or race and ethnicity, without repeating mediator selection within subgroups. Conditional indirect effects and between-group differences were estimated using 2,000 participant-level bootstrap resamples stratified by moderator category. Bootstrap *P*-values for between-group differences were adjusted using BH-FDR within the family of tests corresponding to each moderator.

Sensitivity analyses were restricted to the same candidate pathways. Cross-sectional analyses used fixed-pathway ordinary least squares (OLS) regression, separately adding APOE ε4 carrier status, log-transformed WMH volume, or diabetes, or excluding participants with a history of stroke. Indirect-effect intervals were estimated using 20,000 Monte Carlo simulations. Longitudinal sensitivity models separately included each additional covariate and its interaction with time, or excluded participants with a history of stroke. Additional models included all core covariate-by-time interactions, followed by further adjustment for WMH and WMH-by-time. In each longitudinal sensitivity analysis, models were fitted for all 14 candidate pathways using the corresponding analysis sample. Cluster bootstrap resampling was performed only for the five pathways whose intervals excluded zero in the primary longitudinal analysis. Analyses involving missing data used model-specific complete-case samples; multiple imputation was not performed. Detailed model specifications and computational procedures are provided in the Supplementary Methods.

## 3 Results

### 3.1 Study sample and participant characteristics

The cross-sectional analysis included 2,606 participants, of whom 788 had two eligible cognitive assessments and were included in the longitudinal analysis. The median interval between assessments was 2.17 years (IQR, 2.00–2.34). At baseline, the mean age was 65.0 years (SD, 8.8), and 1,608 participants (61.7%) were women. The sample included 833 non-Hispanic White (32.0%), 931 Hispanic (35.7%), and 842 non-Hispanic Black (32.3%) participants. Hypertension and diabetes were present in 1,747 (67.0%) and 637 (24.4%) participants, respectively. Among the 2,497 participants with APOE data, 753 (30.2%) were APOE ε4 carriers. Baseline characteristics are presented in Table 1.

**TABLE 1:** Baseline characteristics of the analytic sample by availability of longitudinal cognitive assessments.

Characteristic	Overall (*N* = 2,606)	Longitudinal subset (*N* = 788)	No second eligible assessment (*N* = 1,818)	Absolute SMD
Age, years, mean (SD)	65.0 (8.8)	65.3 (8.7)	64.8 (8.8)	0.06
Female, *n* (%)	1,608 (61.7)	507 (64.3)	1,101 (60.6)	0.08
Education, years, mean (SD)	13.4 (4.2)	13.7 (4.1)	13.3 (4.2)	0.11
Race and ethnicity, n (%)				
Non-Hispanic White	833 (32.0)	318 (40.4)	515 (28.3)	0.26
Hispanic	931 (35.7)	269 (34.1)	662 (36.4)	0.05
Non-Hispanic Black	842 (32.3)	201 (25.5)	641 (35.3)	0.21
TyG, mean (SD)	8.60 (0.63)	8.65 (0.62)	8.58 (0.64)	0.10
TyG-WHtR, mean (SD)	5.37 (1.02)	5.38 (1.03)	5.37 (1.01)	0.01
TyG-VAI, median (IQR)	12.81 (7.74–20.79)	13.58 (7.98–22.17)	12.51 (7.65–19.93)	0.05
Hypertension, *n* (%)	1,747 (67.0)	518 (65.7)	1,229 (67.6)	0.04
Current smoking, *n* (%)	208 (8.0)	47 (6.0)	161 (8.9)	0.11
Diabetes, *n* (%)	637 (24.4)	189 (24.0)	448 (24.6)	0.01
History of stroke, *n/N* (%)	106/2,605 (4.1)	35/788 (4.4)	71/1,817 (3.9)	0.03
APOE ε4 carrier, *n/N* (%)	753/2,497 (30.2)	212/774 (27.4)	541/1,723 (31.4)	0.09
Log-transformed WMH volume, median (IQR)	0.69 (0.25–1.50)	0.69 (0.22–1.48)	0.70 (0.26–1.50)	0.04
Vida scanner, *n* (%)	1,623 (62.3)	339 (43.0)	1,284 (70.6)	0.58
DTI–clinical assessment interval, days, median (IQR)	24 (15–34)	22 (14–31)	26 (16–36)	0.31
Cognitive assessment interval, years, median (IQR)	–	2.17 (2.00–2.34)	–	–

Values are mean (SD), median (IQR), or *n* (%), as indicated. Percentages use nonmissing observations; denominators are shown where categorical data are missing. Absolute SMDs compare participants with and without a second eligible assessment and use means and pooled SDs for continuous variables and category-specific proportions for categorical variables. An en dash indicates not applicable. APOE, apolipoprotein E; DTI, diffusion tensor imaging; IQR, interquartile range; SD, standard deviation; SMD, standardized mean difference; TyG, triglyceride–glucose index; TyG-VAI, TyG × visceral adiposity index; TyG-WHtR, TyG × waist-to-height ratio; WMH, white matter hyperintensity.

### 3.2 Associations between insulin-resistance indices and cognitive domains

Of the 18 correlations between the three IR surrogate indices and six cognitive domains, 15 remained statistically significant after both BH-FDR and Bonferroni correction. All 15 correlations were negative (r range, -0.173 to -0.061). The strongest correlation was between TyG-WHtR and working memory (*r* = -0.173; FDR *q* < 0.001). None of the three IR indices was significantly correlated with episodic memory after BH-FDR correction (Supplementary Table 1).

### 3.3 Cross-sectional statistical indirect associations

The 36 cross-sectional HIMA models evaluated 1,152 tract-level indirect associations. HIMA screening with within-model DACT-BH correction identified 14 candidate pathways, all with negative indirect estimates. All 14 were RD pathways, seven for TyG and seven for TyG-WHtR. No candidate pathways were selected for AD or TyG-VAI (Supplementary Table 2).

The 95% conditional Monte Carlo uncertainty intervals excluded zero for 11 of the 14 candidate pathways, including six for TyG-WHtR and five for TyG. The cognitive outcomes were processing speed (four pathways), executive function (three), episodic memory (two), working memory (one), and global cognition (one). Seven pathways involved projection fibers and four the right cingulum (cingulate gyrus portion). The indirect estimate was largest in magnitude for the TyG-WHtR–right cingulum RD–executive function pathway (-0.0093; 95% conditional Monte Carlo uncertainty interval, -0.0160 to -0.0037).

In the supplementary model-level analysis, two models had Simes combined *P*-values with BH-FDR-adjusted *q* < 0.05: TyG–RD–executive function (*q* = 0.0446) and TyG-WHtR–RD–episodic memory (*q* = 0.0456) (Supplementary Table 3).

### 3.4 Bootstrap assessment of HIMA selection stability

All 36,000 HIMA model fits were completed successfully across 1,000 participant-level bootstrap samples. Selection frequencies for the original 14 candidate pathways ranged from 6.7% to 42.0%. The TyG–right posterior limb of the internal capsule RD–working memory pathway had the highest selection frequency (42.0%). The TyG-WHtR–right superior corona radiata RD–processing speed pathway had the lowest frequency (6.7%) (Supplementary Table 4).

### 3.5 Exploratory moderation analyses

Moderation analyses by sex, APOE ε4 carrier status, and race and ethnicity included 70 between-group comparisons of indirect effects. Three comparisons had unadjusted 95% bootstrap intervals that excluded zero. However, none remained statistically significant after BH-FDR correction within the corresponding moderator-specific family of tests (Supplementary Table 5).

### 3.6 Longitudinal follow-up evaluation of cross-sectionally selected pathways

In the longitudinal sample of 788 participants, 95% bootstrap intervals for the indirect estimates excluded zero for five of the 14 cross-sectional candidate pathways. All five estimates were negative, and the cognitive outcomes were processing speed and executive function. Four pathways involved right cingulum RD (cingulate gyrus portion) and one right superior corona radiata RD.

For both TyG and TyG-WHtR, the right cingulum RD pathways corresponded to lower subsequent rates of change in processing speed and executive function. The TyG-WHtR pathway involving right superior corona radiata RD was associated with processing speed change. The TyG-WHtR–right cingulum RD–executive function pathway yielded the largest longitudinal indirect estimate in magnitude: -0.0054 per year (95% bootstrap interval, -0.0110 to -0.0009).

Across all 14 pathways, five DTI-by-time interaction terms remained statistically significant after BH-FDR correction. For four of these pathways, the bootstrap intervals for the indirect estimates also excluded zero. The TyG-WHtR–right superior corona radiata RD–processing speed pathway met the indirect-interval criterion, but its DTI-by-time interaction did not meet the BH-FDR threshold (*q* = 0.0554). The main longitudinal results are presented in Table 2, and results for all 14 pathways are provided in Supplementary Table 6. Cross-sectional and longitudinal indirect estimates are shown in Figure 2.

**TABLE 2:** Cross-sectional and longitudinal indirect estimates for candidate pathways with longitudinal bootstrap intervals excluding zero.

Exposure	Cognitive domain	White matter tract (RD)	Cross-sectional indirect effect (95% CI)	Longitudinal indirect effect/y (95% bootstrap CI)
TyG-WHtR	Processing speed	Right cingulum (cingulate gyrus)	−0.0080 (−0.0140 to −0.0027)	−0.0035 (−0.0066 to −0.0007)
TyG-WHtR	Processing speed	Right superior corona radiata	−0.0037 (−0.0077 to −0.0007)	−0.0021 (−0.0043 to −0.0002)
TyG-WHtR	Executive function	Right cingulum (cingulate gyrus)	−0.0093 (−0.0160 to −0.0037)	−0.0054 (−0.0110 to −0.0009)
TyG	Processing speed	Right cingulum (cingulate gyrus)	−0.0036 (−0.0075 to −0.0007)	−0.0019 (−0.0043 to −0.0004)
TyG	Executive function	Right cingulum (cingulate gyrus)	−0.0039 (−0.0082 to −0.0008)	−0.0030 (−0.0064 to −0.0004)

Shown are five of the 14 cross-sectionally selected pathways whose longitudinal indirect-effect bootstrap intervals excluded zero. All 14 pathways were evaluated without mediator reselection (Supplementary Table 6). Cross-sectional and longitudinal sample sizes were 2,606 and 788, respectively. Models adjusted for age, sex, education, race and ethnicity, hypertension, current smoking, and scanner. MC, Monte Carlo; RD, radial diffusivity; SD, standard deviation; TyG, triglyceride–glucose index; TyG-WHtR, TyG × waist-to-height ratio.

**FIGURE 2:**
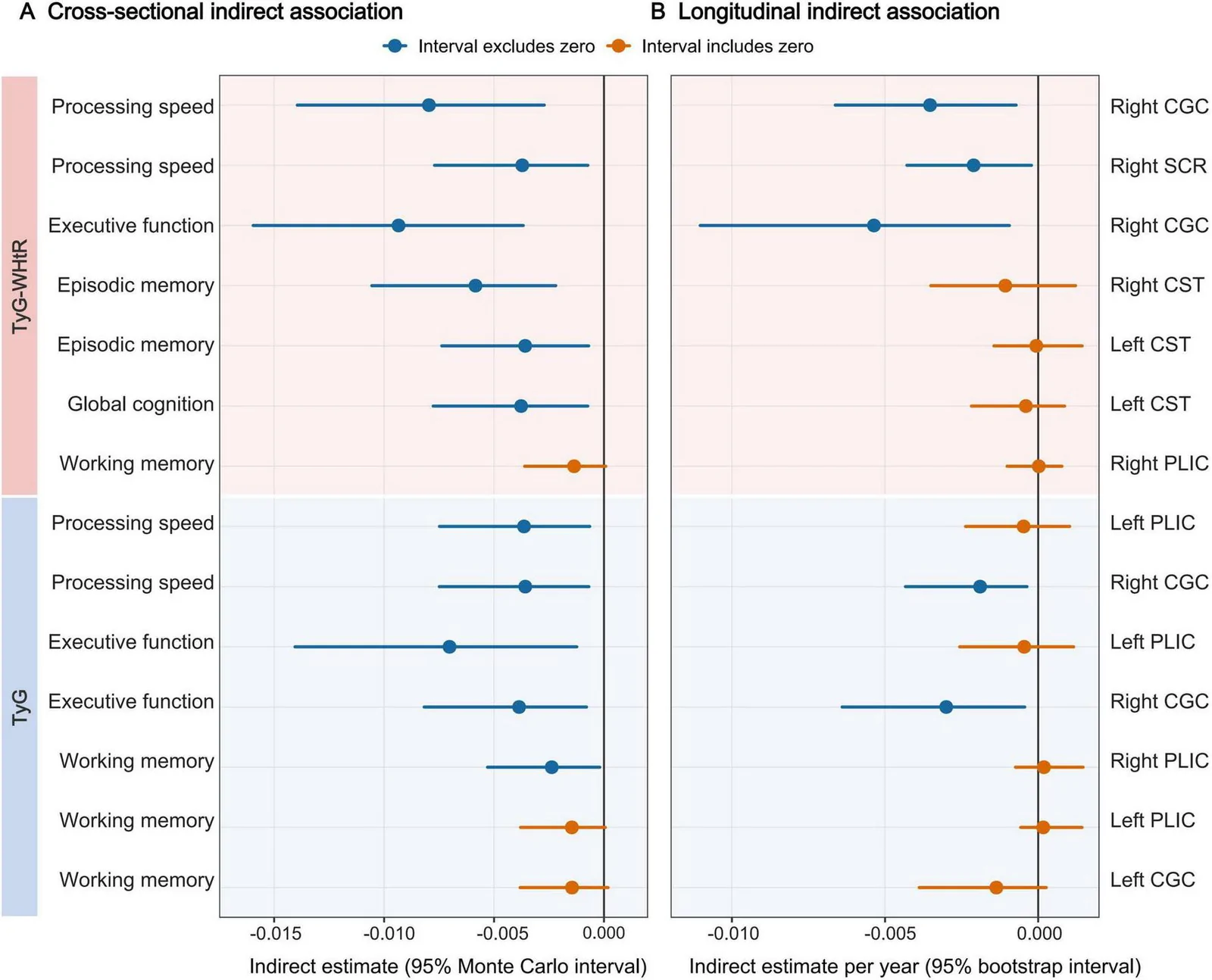
Cross-sectional and longitudinal indirect associations linking metabolic indices, white matter radial diffusivity, and cognition. Points and horizontal bars represent indirect estimates and 95% intervals for all 14 RD pathways selected by cross-sectional HIMA. **(A)** Cross-sectional estimates (*n* = 2,606), with conditional Monte Carlo uncertainty intervals from 20,000 simulations. **(B)** Longitudinal estimates per year (*n* = 788), with percentile intervals from 2,000 participant-level cluster bootstrap samples. All pathways were evaluated longitudinally without mediator reselection. Rows are grouped by metabolic index, with cognitive domains on the left and white matter tracts on the right. Blue and orange indicate intervals excluding and including zero, respectively. Vertical lines mark zero; horizontal scales differ between panels. Intervals do not account for pathway-selection uncertainty, and longitudinal intervals are not adjusted for pathway-level multiplicity. Colors therefore do not indicate FDR-adjusted significance of longitudinal indirect estimates. CGC, cingulum (cingulate gyrus portion); CST, corticospinal tract; FDR, false discovery rate; HIMA, high-dimensional mediation analysis; PLIC, posterior limb of the internal capsule; RD, radial diffusivity; SCR, superior corona radiata; TyG, triglyceride–glucose index; TyG-WHtR, triglyceride–glucose index multiplied by waist-to-height ratio.

### 3.7 Sensitivity analyses

In the cross-sectional fixed-pathway OLS reference models, six of the 14 candidate pathways had 95% Monte Carlo uncertainty intervals that excluded zero. After separate additional adjustment for APOE ε4 carrier status, log-transformed WMH volume, or diabetes, the corresponding counts were six, five, and four. Six pathways had intervals excluding zero after participants with a history of stroke were excluded. The WMH-adjusted analysis included 2,515 participants.

All five pathways whose intervals excluded zero in the primary longitudinal analysis retained this pattern after separate adjustment for APOE ε4 carrier status or diabetes and their respective interactions with time. After adjustment for WMH and WMH-by-time, two of the five pathways had intervals that excluded zero; this analysis included 765 participants. Three retained intervals excluding zero after participants with a history of stroke were excluded.

After adding all core covariate-by-time interactions, two pathways retained bootstrap intervals excluding zero: the right cingulum RD–processing speed pathways for TyG and TyG-WHtR. With further adjustment for WMH and WMH-by-time, only the TyG–right cingulum RD–processing speed pathway retained an interval that excluded zero (Supplementary Table 7).

## 4 Discussion

In the racially and ethnically diverse HABS-HD community cohort, higher IR-related metabolic burden was associated with poorer cognitive performance, with tract-level white matter microstructure as a potential biological link. All 14 candidate indirect pathways selected by cross-sectional HIMA involved RD and either TyG or TyG-WHtR. Conditional Monte Carlo uncertainty intervals excluded zero for 11 pathways, primarily involving projection fibers and the right cingulum. In the longitudinal follow-up analysis, bootstrap intervals for the indirect estimates excluded zero for five pathways, without mediator reselection. These pathways concerned the right cingulum and right superior corona radiata, with cognitive outcomes concentrated in processing speed and executive function. Exploratory analyses found no evidence of moderation by sex, APOE ε4 carrier status, or race and ethnicity after FDR correction. These findings suggest that white matter microstructure may contribute to the association between metabolic burden and cognitive aging.

Previous population-based studies have separately linked IR to white matter microstructural abnormalities and cognitive function. Among cognitively normal middle-aged and older adults, higher homeostasis model assessment of insulin resistance (HOMA-IR) was associated with abnormal diffusion measures in regions including the corpus callosum and corona radiata (Ryu et al., 2014). In 1,709 community-dwelling adults without diabetes, lower insulin sensitivity and higher HOMA-IR were associated with higher MD, AD, and RD in the corona radiata, internal capsule, and corpus callosum (Zhou et al., 2024). Long-term trajectories characterized by high TyG-related index values have also been associated with greater WMH burden and poorer white matter microstructural integrity (Shu et al., 2025). For cognition, higher TyG has been associated with poorer performance among older adults without diabetes (Wei et al., 2023). Higher HOMA-IR and insulin levels have also been associated with lower immediate recall scores in some population groups (He et al., 2021). Longitudinal studies have linked higher TyG to subsequent cognitive decline (Wang K. et al., 2022) and reported a dose–response relationship between TyG-WHtR and incident cognitive impairment (Hou et al., 2024).

Studies directly examining statistical indirect associations between IR and cognition involving white matter imaging measures remain limited. Wang et al. studied 55 patients with type 2 diabetes and 40 controls, assessing IR with the updated homeostasis model assessment (HOMA2-IR). Their analysis identified a statistical indirect association between HOMA2-IR and executive function involving FA in the genu of the corpus callosum (Wang X. et al., 2022). In 110 patients with cerebral small vessel disease but without diabetes, Yang et al. reported an indirect association between HOMA-IR and cognitive performance involving the DTI analysis along the perivascular space (DTI-ALPS) index (Yang et al., 2025). DTI-ALPS captures diffusion characteristics distinct from conventional tract-level tensor measures. Both studies nevertheless suggest that diffusion MRI phenotypes may help clarify the relationship between metabolism and cognition. Our study extends this work to a larger, multiethnic community sample by evaluating three IR-related indices, AD and RD across 32 white matter tracts, and six cognitive domains. It also examines associations between baseline candidate pathways and subsequent cognitive change.

The cross-sectional pathways with supporting interval estimates predominantly concerned processing speed and executive function. This distribution is consistent with the white matter disconnection account of cognitive aging. Processing speed and executive control depend on rapid, coordinated information transfer through frontal–subcortical and other distributed brain networks, making these functions sensitive to white matter connectivity abnormalities. Studies of healthy aging have linked overall integrity across major white matter tracts to information processing speed (Penke et al., 2010). Longitudinal changes in white matter FA have also been coupled with subsequent changes in processing speed (Oschwald et al., 2019). Studies of cerebral small vessel disease have further associated FA and MD abnormalities with impairments in executive function, attention, and global cognition (Jansma et al., 2024; Xie et al., 2022). The right cingulum identified in our longitudinal pathways connects cingulate, medial frontal, parietal, and temporal regions, whereas the right superior corona radiata carries cortical–subcortical connections. This anatomical distribution is consistent with the dependence of processing speed and executive function on distributed white matter connectivity.

Interpretation of individual tract-level findings is also constrained by selection stability and the multiple-testing results. The original 14 candidate pathways were reselected in 6.7%–42.0% of bootstrap samples when the complete HIMA procedure was repeated. Of the 36 models, only TyG–RD–executive function and TyG-WHtR–RD–episodic memory met the supplementary Simes–BH criterion. Although model-level testing and tract-level screening address different hypotheses, these findings together indicate limited support for the reproducibility of individual candidate pathways. The recurrence of the right cingulum across several longitudinal associations merits further investigation. However, the exposures and cognitive outcomes are correlated, and repeated findings within the same cohort do not constitute independent replication.

Of the two DTI measures evaluated, AD and RD, only RD appeared in the 14 selected cross-sectional pathways. Conditional Monte Carlo intervals excluded zero for 11 of these pathways, and longitudinal bootstrap intervals excluded zero for five. RD reflects mean water diffusion perpendicular to the principal fiber direction. Experimental demyelination studies and MRI–histology comparisons suggest that RD may be relatively sensitive to myelin-related changes (Lazari and Lipp, 2021; Song et al., 2005). Oligodendrocytes require sustained energy and lipid supplies to synthesize and maintain myelin and also provide metabolic support to axons (Tepavčević, 2021). IR-related metabolic disturbances, inflammation, and microvascular dysfunction may interfere with oligodendrocyte metabolism and myelin maintenance (Chen et al., 2024; García-García et al., 2022). Population-based myelin water imaging studies have associated higher BMI and waist circumference with lower cerebral white matter myelin water fraction (Bouhrara et al., 2021). Oligodendrocyte injury, myelin breakdown, and cognitive dysfunction have also been observed in animal models of diabetes (Yang et al., 2024). These observations provide biological plausibility for a myelin-related interpretation. However, RD is not specific to myelin and is also influenced by fiber organization, crossing fibers, extracellular water, and inflammation. Our findings are therefore compatible with myelin-related changes but do not provide direct evidence of myelin injury.

Triglyceride–glucose index and TyG-WHtR each accounted for seven of the 14 cross-sectional HIMA candidate pathways. TyG primarily reflects the metabolic burden associated with fasting glucose and triglyceride levels, whereas WHtR adds information on central adiposity (Tahapary et al., 2022). Central adiposity is closely associated with low-grade inflammation, oxidative stress, and endothelial dysfunction, all of which may contribute to cerebral small vessel and white matter injury (García-García et al., 2022; Powell-Wiley et al., 2021). A cross-sectional analysis of 34,653 UK Biobank participants associated measures of overall and central adiposity with greater WMH burden, with the strongest association observed for waist circumference. Among 2,664 participants with repeat imaging, several adiposity measures were also associated with WMH change over time (Wang et al., 2024). Another study with 10 years of follow-up associated persistently high TyG-WHtR trajectories with greater WMH burden and poorer white matter microstructural integrity (Shu et al., 2025). Cohort evidence also supports a dose–response relationship between TyG-WHtR and incident cognitive impairment (Hou et al., 2024). Our TyG-WHtR findings are thus consistent with the hypothesis that central adiposity may increase the risk of white matter injury associated with metabolic burden.

The WMH sensitivity analyses provide additional context for these associations. WMH volume quantifies white matter lesions visible on conventional MRI, whereas DTI can detect microstructural abnormalities within lesions and normal-appearing white matter. These measures may therefore capture partially overlapping changes related to cerebral small vessel disease (Ferris et al., 2025; Voorter et al., 2025). Given this overlap and uncertainty about the causal position of WMH, we included WMH in sensitivity analyses to evaluate DTI-related indirect associations conditional on lesion burden. WMH may be a downstream manifestation of metabolic effects on brain tissue, and adjustment could block some of the pathways of interest (Andrews et al., 2020; Wang et al., 2017). After adding WMH and WMH-by-time, bootstrap intervals continued to exclude zero for two of the original five longitudinal pathways, indicating sensitivity to WMH adjustment. This change may reflect pathological information captured by both WMH and DTI, but it may also reflect changes in confounding control or estimation precision.

This study has several limitations. The observational design and assessment of metabolic and DTI measures near baseline prevented us from establishing the temporal ordering of exposure and mediator. Although cognition was assessed longitudinally, reverse associations and residual confounding cannot be excluded, and the findings should be interpreted as statistical indirect associations. TyG, TyG-WHtR, and TyG-VAI are surrogate indices of IR influenced by glucose and lipid metabolism and body fat distribution, rather than direct measurements of insulin sensitivity. The longitudinal analysis included only two cognitive assessments, separated by a median of 2.17 years. This limited our ability to characterize nonlinear trajectories, and the scores may have been affected by practice effects. The absence of repeated metabolic and DTI measurements also limited the assessment of dynamic mediation processes.

Statistical inference was further limited by using the same cross-sectional sample for candidate pathway selection and Monte Carlo interval estimation. The resulting intervals did not account for additional uncertainty arising from selection. Repeating the complete HIMA procedure in bootstrap samples assessed selection stability but did not resolve post-selection interval inference. The longitudinal analysis also used participants from the same cohort, and its pathway-specific bootstrap intervals were not adjusted for multiplicity across pathways. BH-FDR adjustment of b_time *P*-values applied only to the DTI-by-time interaction terms.

The longitudinal sample represented only a subset of the baseline cohort, and analyses incorporating additional covariates used complete-case samples. These analyses may therefore be subject to selection bias related to follow-up availability and missing data. Independent cohorts with longer follow-up and repeated metabolic and imaging measurements would help assess the reproducibility and temporal ordering of these candidate associations.

## 5 Conclusion

In the multiethnic HABS-HD community cohort, white matter RD was involved in statistical indirect associations of TyG and TyG-WHtR with cognitive performance. Some baseline pathways were also associated with subsequent changes in processing speed and executive function. Selection stability was limited for individual pathways, and longitudinal associations were sensitive to adjustment for WMH and covariate-by-time interactions. These findings add evidence linking metabolic burden, white matter microstructure, and cognitive aging.

## Data Availability

The data analyzed in this study were obtained from HABS-HD Releases 7 and 8. Access to these data is subject to the applicable HABS-HD data-access and approval procedures.

## The Health and Aging Brain Study (HABS-HD) Study Team

HABS-HD MPIs: Sid E O’Bryant, Kristine Yaffe, Arthur Toga, Robert Rissman, & Leigh Johnson; and the HABS-HD Investigators: Meredith Braskie, Kevin King, James R Hall, Melissa Petersen, Raymond Palmer, Robert Barber, Yonggang Shi, Fan Zhang, Rajeh Nandy, Roderick McColl, David Mason, Bradley Christian, Nicole Phillips, Stephanie Large, Joe Lee, Badri Vardarajan, Monica Rivera Mindt, Amrita Cheema, Lisa Barnes, Mark Mapstone, Annie Cohen, Amy Kind, Ozioma Okonkwo, Raul Vintimilla, Zhengyang Zhou, Michael Donohue, Rema Raman, Matthew Borzage, Michelle Mielke, Beau Ances, Ganesh Babulal, Jorge Llibre-Guerra, Carl Hill and Rocky Vig.
