# Supplementary material for: Insulin resistance–related metabolic burden, white matter microstructure, and cognitive change: the HABS-HD study
Source: Front Neurosci. 2026 Oct 1;20:1967890. doi: 10.3389/fnins.2026.1967890 (PMC13668860; doi:10.3389/fnins.2026.1967890)
Supplement: Supplementary file 1 [file Supplementary_file_1.docx]

**Supplementary Statistical Methods**

**Statistical software and descriptive analyses**

Statistical analyses were performed in R 4.6.0. The principal packages were HIMA 2.3.3, ncvreg 3.16.0, lme4 2.0-1, lmerTest 3.2-1, future 1.70.0, and furrr 0.4.0. Continuous variables were summarized using means, standard deviations, medians, and the 25th and 75th percentiles; categorical variables were summarized as counts and percentages. Unless otherwise specified, tests were two-sided, with P values or false discovery rate (FDR)-adjusted q values below 0.05 used as statistical evidence thresholds.

Eighteen unadjusted Pearson correlations were calculated between the three insulin resistance (IR) surrogate indices and six cognitive domains. Correlation coefficients, Fisher z-based 95% confidence intervals, and unadjusted P values were reported. Benjamini–Hochberg (BH) and Bonferroni corrections were applied separately across all 18 tests.

**Variable scaling and covariate coding**

IR indices were standardized using baseline means and sample standard deviations. Tract-level DTI measures were obtained from the standardized preprocessing outputs. Cognitive test scores were oriented so that higher scores indicated better performance, and the six domains were constructed as described in the main Methods. Multitest domains were averages of the corresponding test z scores and were not subsequently rescaled to unit variance.

Core covariates were age, sex, education, race and ethnicity, hypertension, current smoking, and scanner. Race and ethnicity were represented by two indicators, with non-Hispanic White participants as the reference group. Sex, hypertension, current smoking, and scanner were coded as binary variables. During HIMA fitting, exposures, mediators, and numerically coded covariates were centered and divided by their sample standard deviations. Cognitive domains retained their established scoring scales.

**Cross-sectional HIMA analysis**

The three IR indices, two DTI measures, and six cognitive domains defined 36 exposure–DTI measure–cognitive domain models. Each model evaluated 32 tracts for one DTI measure; axial diffusivity (AD) and radial diffusivity (RD) were modeled separately. A local implementation using HIMA 2.3.3 components combined marginal screening, minimax concave penalty (MCP) regression, ordinary least squares (OLS) refitting, and DACT composite-null testing.

Marginal screening ranked mediators by the absolute product of covariate-adjusted exposure–mediator and mediator–outcome coefficients. The retention count was twice the sample size divided by its natural logarithm, rounded upward and capped at 32. This retained all 32 tracts at the observed sample size. Retained mediators, the exposure, and covariates were then entered into Gaussian MCP regression using the default ncvreg penalty settings. The penalty parameter was selected by the Bayesian information criterion.

For each tract, the a path was estimated by regressing its DTI measure on the exposure and core covariates using OLS. For mediators with nonzero MCP coefficients, b-path estimates and standard errors came from an OLS model containing those mediators, the exposure, and core covariates. Each mediator not retained by MCP was added individually to that model to estimate its b path. Thus, component-path coefficients and standard errors were calculated for all 32 tracts; unselected mediators were not assigned P values of 1. The statistical indirect estimate was defined as the product of the a- and b-path coefficients.

Two-sided component-path P values were calculated from coefficient-to-standard-error ratios using a standard normal approximation and supplied to DACT. DACT evaluated the composite null hypothesis that at least one component-path coefficient was zero. The 32 DACT P values within each model underwent standard BH step-up adjustment. Pathways with q values at or below 0.05 defined the original cross-sectional candidate set. Subsequent interval estimation, stability assessment, and follow-up analyses did not modify this set.

**Conditional Monte Carlo uncertainty intervals**

For each original candidate pathway, 20,000 Monte Carlo simulations used the OLS estimates and standard errors described above. Each simulation independently sampled the two component coefficients from normal distributions centered on their estimates, with standard deviations equal to their respective standard errors. The sampled coefficients were multiplied, and the 2.5th and 97.5th percentiles of the resulting products formed the 95% uncertainty interval. Independent sampling omitted covariance between the two component-path estimates.

“Conditional” denotes treating the selected model and candidate set as fixed, rather than performing formal selective inference. Because selection and estimation used the same dataset, these intervals omitted additional uncertainty arising from mediator selection. They were not interpreted as selection-adjusted confidence intervals. Interval exclusion of zero was not used to further select candidate pathways.

**Model-level multiplicity analysis**

Within each HIMA model, the 32 DACT P values were ordered from smallest to largest. Each ordered P value was multiplied by 32 and divided by its rank. The minimum resulting value, capped at 1, formed the model-level Simes P value. The resulting 36 P values underwent BH-FDR adjustment.

This analysis provided supplementary evidence at the exposure–DTI measure–cognitive domain level and did not impose post hoc gating on tract-level candidates. Within-model BH and model-level Simes–BH addressed separate testing levels; they did not constitute a single global FDR adjustment across all 1,152 tract-level tests.

**Bootstrap assessment of mediator-selection stability**

Baseline participants were sampled with replacement 1,000 times, with each bootstrap sample containing the same number of participants as the original sample. All 36 HIMA models were rerun in each sample. This included within-sample scaling, marginal screening, MCP fitting, OLS refitting, DACT testing, and within-model BH adjustment. Resampling started from the existing analysis dataset without repeating upstream imaging preprocessing or estimating new cognitive reference parameters.

Selection frequency was calculated for each exact combination of exposure, DTI measure, tract, and cognitive domain. Its denominator was the number of successful bootstrap fits for the corresponding model. Selection counts, frequencies, binomial-approximation Monte Carlo standard errors, and 95% Wilson intervals were reported. These measures describe simulation precision from a finite number of bootstrap samples, not the probability of replication in an independent cohort.

No post hoc stability threshold was imposed, and selection frequencies did not modify the original candidate set. Pathways selected only in bootstrap samples were not added to the primary cross-sectional or longitudinal analyses.

**Exploratory moderation analyses**

Moderation analyses were restricted to the original cross-sectional candidates and separately evaluated sex, APOE ε4 carrier status, and race and ethnicity in pooled samples. Mediators were not reselected within subgroups. Each pathway was evaluated using single-mediator OLS models. The a-path model included the exposure, moderator, and exposure-by-moderator interaction. The b-path model included the exposure, mediator, moderator, exposure-by-moderator interaction, and mediator-by-moderator interaction. Both models included the corresponding core covariates.

When sex or race and ethnicity served as the moderator, its main effect was represented by the moderator term rather than duplicated among covariates. Sex contrasts were female minus male, and APOE ε4 contrasts were carrier minus noncarrier. Race and ethnicity contrasts were non-Hispanic Black minus non-Hispanic White, Hispanic minus non-Hispanic White, and Hispanic minus non-Hispanic Black.

Conditional a and b coefficients and their products were estimated at each moderator level. Between-group differences were calculated by subtracting the corresponding conditional products. Participant-level nonparametric bootstrap resampling was performed 2,000 times within moderator categories, preserving category-specific sample sizes. Intervals used the 2.5th and 97.5th percentiles of finite bootstrap estimates.

Two-sided bootstrap P values were calculated from the proportions of estimates on either side of zero. Counts at or below zero and at or above zero were each increased by one and divided by the number of valid replicates plus one. The smaller adjusted proportion was doubled and capped at 1.

Between-group P values underwent BH-FDR adjustment within moderator-specific families comprising 14 sex, 14 APOE ε4, and 42 race and ethnicity comparisons. Exploratory moderation evidence required both an interval excluding zero and a q value below 0.05 within the corresponding family. Moderation results did not alter the primary candidate set.

**Longitudinal follow-up analysis**

All original cross-sectional candidate pathways were carried forward without mediator reselection. Exposures, DTI mediators, and covariates were fixed at baseline values across visits. Individual cognitive tests at both visits were standardized using test-specific means and standard deviations from the full baseline sample. Cognitive domains were then constructed using identical scoring rules. Follow-up time was the number of days since the baseline cognitive visit divided by 365.25.

For each pathway, the a coefficient was reestimated among participants with two valid cognitive assessments who entered the corresponding longitudinal model. This OLS model regressed the baseline DTI mediator on the baseline IR index and core covariates.

Cognitive change was evaluated using linear mixed-effects models, each containing only the DTI mediator for the current pathway. Fixed effects included time, baseline IR, baseline DTI, their respective interactions with time, and core covariates. Models included participant-specific random intercepts without random slopes and were fitted by maximum likelihood. Fixed-effect P values used the default Satterthwaite degrees-of-freedom approximation in lmerTest.

The DTI-by-time coefficient, denoted b_time, represented the association between baseline DTI and subsequent cognitive change rate. The longitudinal indirect estimate was the product of the reestimated a coefficient and b_time. Each pathway underwent 2,000 participant-cluster bootstrap resamples, jointly reestimating both coefficients and their product. All repeated cognitive observations were retained for each sampled participant. Repeated draws of the same participant received distinct bootstrap cluster identifiers. Variable-scaling parameters remained fixed during longitudinal resampling.

The primary longitudinal analysis reported 95% percentile intervals only when at least 95% of replicates returned nonmissing products. Intervals excluding zero were treated as descriptive follow-up evidence, not as pathway-level multiplicity-adjusted significance. The b_time P values underwent BH-FDR correction across all 14 candidates. This adjustment applied only to the DTI-by-time component and did not adjust inference for the indirect products.

Because exposures and DTI mediators were measured at or near baseline, the analysis did not establish their temporal ordering. It was not interpreted as a longitudinal causal mediation model with time-varying exposures and mediators.

**Sensitivity analyses**

All sensitivity analyses used the original cross-sectional candidate set without reselection. Cross-sectional analyses first fitted single-mediator OLS reference models containing core covariates. Separate models additionally adjusted for APOE ε4 carrier status, log-transformed WMH volume, or diabetes, or restricted the sample to participants without prior stroke. Both component paths used the same model-specific complete-case sample, and additional covariates entered both models. Exposures, mediators, and covariates were restandardized within each sample, whereas cognitive domains retained their original scales. Conditional intervals used 20,000 independent normal Monte Carlo draws.

These single-mediator models did not retain the other MCP-selected mediators from HIMA. Sensitivity comparisons therefore used the fixed-pathway OLS reference rather than requiring estimates or intervals to reproduce the HIMA refits.

Longitudinal sensitivity models separately added APOE ε4 status, diabetes, or WMH and the corresponding interaction with time, or excluded participants with prior stroke. Additional main effects also entered the a-path model, and both component paths were reestimated within the same model-specific sample. Further models included all core covariate-by-time interactions, followed by additional adjustment for WMH and WMH-by-time.

WMH analyses used the log-transformed volume supplied in the source report; the analysis code did not recalculate its logarithmic transformation. Longitudinal models additionally standardized this variable using available baseline values and retained that scale during resampling. WMH adjustment assessed sensitivity of indirect estimates and uncertainty to lesion burden. It did not establish pathological independence or overlap between WMH and DTI.

All 14 candidates received point estimates under each longitudinal sensitivity specification. Cluster bootstrap resampling, with 2,000 replicates per pathway, was restricted to the five pathways whose primary longitudinal intervals excluded zero. Retention required an unchanged indirect-estimate direction and a 95% percentile interval excluding zero. Pathways without bootstrap reassessment were not classified as failing retention.

**Missing data and computational reproducibility**

Primary cross-sectional analyses required complete exposure, cognitive domain, core covariate, and DTI data. Moderation and additional-covariate analyses used model-specific complete-case samples. Longitudinal analyses required two eligible cognitive assessments and complete variables for the corresponding model. Multiple imputation was not performed.

Random analyses used fixed seeds. The base seed was 20260711 for cross-sectional Monte Carlo and longitudinal bootstrap analyses, 20260710 for moderation, and 20260904 for HIMA stability assessment. Parallel bootstrap analyses used reproducible random-number settings with pathway-, model-, or replicate-specific seed offsets.

HIMA stability analyses recorded model errors and model-specific successful replicate counts. Moderation summaries used finite bootstrap estimates. Longitudinal bootstrap success was defined by a nonmissing product; convergence warnings and singularity were not separate replicate-exclusion criteria. Singularity was recorded separately for observed mixed models. Saved primary longitudinal results and bootstrapped sensitivity models each contained 2,000 nonmissing products per evaluated pathway.

The workflow retained input checksums, software versions, model definitions, random seeds, computational logs, bootstrap estimates, and available diagnostic records. These records and the analysis code provide an auditable account of the implemented procedures.
